# Pesticides in house dust from urban and farmworker households in California: an observational measurement study

**DOI:** 10.1186/1476-069X-10-19

**Published:** 2011-03-16

**Authors:** Lesliam Quirós-Alcalá, Asa Bradman, Marcia Nishioka, Martha E Harnly, Alan Hubbard, Thomas E McKone, Jeannette Ferber, Brenda Eskenazi

**Affiliations:** 1 Center for Environmental Research and Children's Health (CERCH), School of Public Health, University of California, 1995 University Avenue Suite 265, Berkeley, CA 94704, USA; 2 Battelle Memorial Institute, 505 King Avenue, Columbus, OH 43201, USA; 3 California Department of Public Health, Environmental Health Investigations Branch, 850 Marina Bay Parkway P-3, Richmond, CA 94804, USA; 4 Division of Biostatistics, School of Public Health, University of California, Berkeley 50 University Hall, MC 7356, Berkeley, CA 94720, USA; 5 Lawrence Berkeley National Laboratory, One Cyclotron Road, Mail stop 90R3058, Berkeley, CA 95720, USA

## Abstract

**Background:**

Studies report that residential use of pesticides in low-income homes is common because of poor housing conditions and pest infestations; however, exposure data on contemporary-use pesticides in low-income households is limited. We conducted a study in low-income homes from urban and agricultural communities to: characterize and compare house dust levels of agricultural and residential-use pesticides; evaluate the correlation of pesticide concentrations in samples collected several days apart; examine whether concentrations of pesticides phased-out for residential uses, but still used in agriculture (i.e., chlorpyrifos and diazinon) have declined in homes in the agricultural community; and estimate resident children's pesticide exposures via inadvertent dust ingestion.

**Methods:**

In 2006, we collected up to two dust samples 5-8 days apart from each of 13 urban homes in Oakland, California and 15 farmworker homes in Salinas, California, an agricultural community (54 samples total). We measured 22 insecticides including organophosphates (chlorpyrifos, diazinon, diazinon-oxon, malathion, methidathion, methyl parathion, phorate, and tetrachlorvinphos) and pyrethroids (allethrin-two isomers, bifenthrin, cypermethrin-four isomers, deltamethrin, esfenvalerate, imiprothrin, permethrin-two isomers, prallethrin, and sumithrin), one phthalate herbicide (chlorthal-dimethyl), one dicarboximide fungicide (iprodione), and one pesticide synergist (piperonyl butoxide).

**Results:**

More than half of the households reported applying pesticides indoors. Analytes frequently detected in both locations included chlorpyrifos, diazinon, permethrin, allethrin, cypermethrin, and piperonyl butoxide; no differences in concentrations or loadings were observed between locations for these analytes. Chlorthal-dimethyl was detected solely in farmworker homes, suggesting contamination due to regional agricultural use. Concentrations in samples collected 5-8 days apart in the same home were strongly correlated for the majority of the frequently detected analytes (Spearman ρ = 0.70-1.00, p < 0.01). Additionally, diazinon and chlorpyrifos concentrations in Salinas farmworker homes were 40-80% lower than concentrations reported in samples from Salinas farmworker homes studied between 2000-2002, suggesting a temporal reduction after their residential phase-out. Finally, estimated non-dietary pesticide intake for resident children did not exceed current U.S. Environmental Protection Agency's (U.S. EPA) recommended chronic reference doses (RfDs).

**Conclusion:**

Low-income children are potentially exposed to a mixture of pesticides as a result of poorer housing quality. Historical or current pesticide use indoors is likely to contribute to ongoing exposures. Agricultural pesticide use may also contribute to additional exposures to some pesticides in rural areas. Although children's non-dietary intake did not exceed U.S. EPA RfDs for select pesticides, this does not ensure that children are free of any health risks as RfDs have their own limitations, and the children may be exposed indoors via other pathways. The frequent pesticide use reported and high detection of several home-use pesticides in house dust suggests that families would benefit from integrated pest management strategies to control pests and minimize current and future exposures.

## Background

Young children are particularly vulnerable to adverse health effects that may result from pesticide exposures. For example, in utero and/or postnatal chronic exposures to organophosphorous (OP) pesticides have been associated with poorer neurodevelopment in children [1-5], and altered fetal growth [6], and shortened gestational duration [7]. Animal studies have also shown that neonatal exposures to other contemporary-use pesticides such as pyrethroids are associated with impaired brain development [8], changes in open-field behaviors, and increased oxidative stress [9].

Pesticides have been measured in residential environments, most notably in indoor dust [10-17]. Poor housing conditions in low-income homes, such as overcrowding and housing disrepair, are associated with pest infestations and increased home pesticide use in both urban and agricultural communities [18,19], potentially increasing pesticide residues indoors. Additionally, the presence of farmworkers in the home and/or proximity of homes to nearby fields in agricultural communities have been associated with higher indoor pesticide concentrations [13,20].

Several studies indicate that pesticide residues persist indoors due to the lack of sunlight, rain, temperature extremes, microbial action, and other factors that facilitate degradation [15]. Semi- and non-volatile pesticides (e.g., OPs and pyrethroids) have chemical properties that increase binding affinity for particles and the tendency to adsorb onto household surfaces such as carpet or dust, also prolonging their persistence indoors [11]. For example, pyrethroid pesticides have low vapor pressures, and high octanol/water (K_ow_) and water/organic carbon (K_oc_) partition coefficients which facilitate partitioning into lipids and organic matter and binding to particulate matter in dust [21]. Because of this, several studies suggest that house dust is an important pathway of pesticide exposure for children [11,15,17,22]. Young children are particularly vulnerable to inadvertent ingestion of pesticide-contaminated dust due to their frequent hand-to-mouth activity and contact with indoor surfaces [15].

California (CA) has intense agricultural pesticide use [23], including OP insecticides. Due to their potential health effects in children, formulations of the OP insecticides, chlorpyrifos and diazinon, were voluntarily phased out for residential uses between 2001 and 2004 [24,25]. One study showed that this residential phase-out resulted in decreased air concentrations among low-income households in New York City [26]. However, these OPs are still used in agriculture and trends in residential contamination of these compounds have not been studied in agricultural communities, where pesticide drift and transport from fields on work clothing may impact indoor pesticide concentrations [20]. It is also widely accepted that house dust is a reservoir for environmental contaminants with concentrations remaining fairly stable [11]; however, to our knowledge, only one study [27] has documented the temporal stability of pesticides in house dust focusing on the OP pesticide chlorpyrifos. Additionally, exposure data on other contemporary-use pesticides (e.g., pyrethroids) in low-income households is limited. In this study, we characterized and compared house dust levels of agricultural and residential-use pesticides from low-income homes in an urban community (Oakland, CA) and an agricultural community (Salinas, CA). We evaluated the correlation of several semi- and non-volatile pesticide concentrations in samples collected several days apart from the same general area in the home; and examined whether house dust concentrations of chlorpyrifos and diazinon declined in Salinas, CA after the U.S. Environmental Protection Agency's (EPA) voluntary residential phase-out of these compounds. Finally, we estimated resident children's potential non-dietary ingestion exposures to these indoor contaminants to determine if exposures via this pathway exceeded current U.S. EPA recommended guidelines.

## Methods

### Study Population

Study participants included families with children between 3 and 6 years of age who were participating in a 16-day biomonitoring exposure study (to be presented elsewhere) conducted during July through September 2006. Through community health clinics and organizations serving low-income populations, we recruited a convenience sample of 20 families living in Oakland, CA, (an urban community in Alameda county) and 20 families living in Salinas, CA (an agricultural community with intense agricultural pesticide use in Monterey county). Participating families were Mexican American or Mexican immigrants and all Salinas households included at least one household member who worked in agriculture. The University of California, Berkeley Committee for the Protection of Human Subjects approved all study procedures and we obtained written informed consent from parents upon enrollment.

### Data Collection

After obtaining informed consent from parents, bilingual staff administered a validated questionnaire [10] to ascertain demographic information on the children and household members, as well as information on factors potentially related to pesticide exposures such as: the presence of pest infestations and storage and use of pesticides in the previous "0-6 days", "7-30 days", "31-90 days" and " >90 days". We also conducted a home inspection to obtain information on housing quality and residential proximity to the nearest agricultural field or orchard. On dust collection days, parents were also asked if any pesticide applications had occurred in/around the home in the preceding 24 hours.

### Dust Sample Collection

Using standard protocols [28], we collected dust samples from an area 1 to 2 m^2 ^with a High Volume Small Surface Sampler (HVS3) which collects particles >5 μm. Most dust samples were collected from carpets where parents indicated children spent time playing, except for two homes with no carpets or rugs, for which we collected samples from upholstered furniture using an attachment on the HVS3. To assess the consistency of concentrations within homes, we collected up to two dust samples, 5-8 days apart, from the same general location in each home. Dust samples were then manually sieved to obtain the fine fraction (<150 μm), which is more likely to adhere to human skin [15]. This fraction was stored at -80°C prior to shipment to Battelle Memorial Institute in Columbus, Ohio for laboratory analysis.

### Laboratory Analysis

Of the 40 homes sampled, 15 Salinas farmworker and 13 Oakland urban homes had sufficient sample mass (≥ 0.5 g) for analysis after measurement of other analytes (to be presented elsewhere). We analyzed two dust samples per home except for one home in each location from which one sample was analyzed, yielding a total of 54 dust samples. For this study, a total of 25 analytes were measured in every sample. Analytes measured included the OP insecticides chlorpyrifos, diazinon, malathion, methidathion, methyl parathion, phorate, tetrachlorvinphos, and one oxidation product of diazinon, diazinon-oxon; the pyrethroid insecticides bifenthrin, allethrin (two isomers), cypermethrin (four isomers), cis- and trans-permethrin, deltamethrin, esfenvalerate, imiprothrin, and prallethrin; the pesticide synergist commonly added to pyrethroid formulations piperonyl butoxide; the herbicide chlorthal-dimethyl; and the fungicide iprodione. We selected target analytes based on regional agricultural and non-agricultural use as reported in the California Department of Pesticide Regulation Pesticide Use Reporting database [29], active ingredients in pesticides used or stored indoors, detection in our prior studies [10,13], and laboratory feasibility. Select physico-chemical properties of the target analytes and information on county-level agricultural and non-agricultural pesticide use in both study locations are provided in the Additional files section (Additional file 1 Table A1).

To measure analytes, we modified a previously published laboratory method [10,13]. Briefly, 0.5 g dust aliquots were fortified with 250 ng of two surrogate recovery standards (SRSs)--fenchlorphos and ^13^C_12_-trans-permethrin--and extracted using ultrasonication in 1:1 hexane:acetone. We used solid phase extraction for sample cleanup, concentrated extracts to 1 mL and then fortified them with an internal standard, dibromobiphenyl. Concentrated extracts were analyzed with an electron impact gas chromatrography mass spectrometer in the multiple ion detection mode (Phenomenex ZB-35 column, 30 m × 0.25 mm ID, 0.25 μm film) with temperatures programmed from 130-340°C at 6°C/min. For each sample analysis set, we analyzed seven calibration curve solutions ranging from 2 to 750 ng/mL (five times higher for deltamethrin) and used a linear least squares regression and the internal method of quantification to prepare calibration curves. A solvent method blank, matrix spike sample (spike = 250 ng), and duplicate study sample were included in each sample analysis set for quality assurance and quality control purposes. We also determined the relative percent difference of the duplicate samples for each analyte measured to ensure that the analytical precision was within acceptable limits.

No analytes were detected in the four solvent method blanks, indicating no laboratory contamination. Analyte recoveries in four randomly-selected matrix spike samples averaged 117 ± 19% for OP analytes, 115 ± 16% for pyrethroid analytes, 82 ± 5% for chlorthal-dimethyl, 112 ± 14% for iprodione; and average SRS recoveries were 113 ± 6% and 128 ± 5% for fenchlorphos and ^13^C_12_-trans-permethrin, respectively. The average relative percent difference in concentration for the 12 analytes detected in duplicate samples was 14 ± 18% (n = 43 difference values spread across 12 analytes), indicating good analytical precision.

### Data Analysis

We first summarized demographic characteristics and computed descriptive statistics for all analytes by location. For subsequent analyses, we focused on analytes frequently detected (i.e., detection frequencies, DF ≥50%). Concentrations below the limit of detection (LOD) were assigned a value of LOD/√2 [30] and results were considered significant at p < 0.05.

We used Fisher's Exact tests to determine if analyte detection frequencies differed between locations. To assess differences in concentrations between study locations, we used linear regression models with a generalized estimating equations (GEE) approach [31] in order to report robust inference that accounts for the non-independence of repeated samples within households. Given the limited number of homes sampled and the homogeneity of the study population, we excluded demographic characteristics as covariates in GEE models. We also examined location differences using analyte loadings, ng/m^2 ^[21]. We calculated loadings by multiplying analyte concentrations by the sieved fine mass and dividing by the area sampled.

To determine the correlation of analyte concentrations between the first and second collections, we computed Spearman rank-order correlations.

To examine temporal trends of chlorpyrifos and diazinon concentrations in farmworker homes after the residential phase-out, we used Wilcoxon Mann-Whitney tests to compare dust concentrations in the 15 (n = 29 samples) Salinas farmworker households sampled in 2006 from our present study with dust concentrations from a subset of 82 Salinas farmworker homes of participants in the CHAMACOS study [13] sampled between 2000 and 2002 (2000, n = 33; 2001, n = 36; 2002, n = 13), and 20 similar households sampled by Bradman et al. [10] in 2002. The same laboratory (Battelle Memorial Institute) and collection methods were used in all studies. In addition, we restricted comparisons to those study homes located in the same zip codes as the homes in the present study. If multiple dust samples were available from any of the study homes in the same year, including the present study, the mean analyte dust concentration was used in our analyses. There were no demographic or household differences between our previous studies and the present study; e.g., all households had at least one farmworker residing in the home and study participants generally represented the farmworker population in Salinas Valley: primarily Mexican or of Mexican descent; Spanish-speaking; low literacy; low income; and frequently reported pesticide applications in the home and wearing work clothes and shoes indoors. Homes were also located >200 feet from the nearest agricultural field. Using the California Department of Pesticide Regulation Pesticide Use Reporting (PUR) database [29], we also computed county-level agricultural and non-agricultural usage of these OP pesticides during 1999-2007 to determine whether temporal changes in residential dust concentrations were concurrent with regional use patterns. Non-agricultural uses included applications for landscape maintenance, public health, commodity fumigation, rights-of-way, and structural pest control applications by licensed applicators which are reported to the state.

Finally, to determine if exposures via the non-dietary ingestion pathway exceed U.S. EPA guidelines for the children in the present study, we calculated hazard quotients (HQ) for the majority of the detected analytes. We focused on the children given their unique vulnerabilities to environmental toxicants [32]. We calculated the HQ as the ratio of the child's potential daily toxicant intake at home via non-dietary ingestion (mg/kg/day) to the specific toxicant chronic reference dose, RfD, (mg/kg/day). The potential daily toxicant intake was calculated as follows:

where C_dust _is the analyte dust concentration in the child's home (mg/g), IR is the dust intake rate--assumed to be 0.10 g/day (100 mg/day) [33], and BW_child _is the child's body weight (kg) obtained at the initial visit. We used chronic RfDs because children ingest small amounts of dust every day [33]. Chronic oral RfDs were available for 14 of the detected pesticides. For those pesticides that have been re-registered in response to the Food Quality Protection Act [34], chronic population adjusted doses (cPAD) were used as the reference dose. An HQ >1.0 would suggest that the child's exposure via non-dietary ingestion, independent of other exposure routes, may exceed the U.S. EPA's RfD.

All statistical analyses were performed using Stata 10 for Windows (StataCorp, College Station, TX).

## Results

### Household demographics and pesticide use

Except for farmworker status, demographic characteristics were similar in both study locations (Table 1). Participating households were within 200% of the poverty line and approximately 50% or more of the homes had at least six household members. Although not statistically significant, pest sightings were more commonly reported in Oakland urban homes compared to Salinas farmworker homes. Most participants reported using pesticides indoors in the three months preceding the study (67% and 85% of farmworker and urban homes, respectively) and the most common location of use was the kitchen. Hand-held pyrethroid sprays were the most common formulation and application method in both locations; applications were mostly targeted at ants and cockroaches. Participants from three homes (one Salinas farmworker home and two Oakland urban homes) reported applying pyrethroid insecticides between the two sampling dates. No products with OP insecticides were stored or reported applied in the homes, at the workplace or on pets. Most participants from Salinas households (80%) reported that farmworkers residing in the home wore their work clothing indoors and about half of them also wore their work shoes indoors. Approximately 27% of Salinas farmworker homes were located <1/4 mile from the nearest agricultural field or orchard.

**Table 1:** Demographic and household characteristics for study participants from farmworker homes in Salinas, CA and urban homes in Oakland, CA.^a^

	Salinas farmworker homes (n = 15)		Oakland urban homes (n = 13)	
	**n**	**(%)**	**n**	**(%)**

Maternal education (highest grade completed)				

< completed 9^th ^grade or lower	8	(53.3)	8	(61.5)

Grades 10-12 (no diploma)	3	(20.0)	1	(7.7)

High school diploma/GED or technical school	2	(13.3)	4	(30.8)

College graduate	2	(13.3)		---

Paternal education (highest grade completed)				

< completed 9 ^th ^grade or lower	12	(85.7)	10	(83.3)

Grades 10-12 (no diploma)	1	(7.1)	1	(8.3)

High school diploma/GED or technical school	1	(7.1)	1	(8.3)

College graduate		---		---

Family income relative to federal poverty level^b^				

≤ Poverty level	10	(66.7)	9	(69.2)

> Poverty level but <200% poverty level	5	(33.3)	4	(30.8)

Number of household members				

3-5	8	(53.3)	5	(38.5)

> 6	7	(46.7)	8	(61.5)

Reported rodent sighting in the home				

Yes	2	(13.3)	3	(23.1)

No	13	(86.7)	10	(76.9)

Reported cockroach sighting in the home				

Yes	3	(20.0)	5	(38.5)

No	12	(80.0)	8	(61.5)

Reported pesticide application in the last 3 months				

Yes	10	(66.7)	11	(84.6)

No	5	(33.3)	2	(15.4)

Farmworkers wore work clothing indoors^c^				

Yes	12	(80.0)		---

No	2	(20.0)		

Farmworkers wore work shoes indoors^c^				

Yes	7	(50.0)		---

No	7	(50.0)		

Farmworkers living in the home (past 3 months)				

0		---	11	(84.6)^d^

1-3	15	(100.0)	2	(15.4)

Farmworkers currently living in the home				

0	1	(6.7)		---

1-3	11	(73.3)		---

4-7	3	(20.0)		---

Distance of home to nearest field/orchard				

50-20 feet	1	(6.7)		---

> 200 feet-1/4 mile	3	(20.0)		---

> 1/4 mile	11	(73.3)		---

a. No statistically significant differences were observed between locations for demographic factors unrelated to farmworker status. b. Families' poverty levels were based on U.S. Department of Health and Human Services thresholds for 2006. Source: http://aspe.hhs.gov/POVERTY/06poverty.shtml. c. One participant in the Salinas group reported that the father was a farmworker during the eligibility screening; however, the father was not living in the home during the sample collection period so information is only available for 14 of the 15 farmworker households for this demographic characteristic. d. Two participants reported having a parent or parent's sibling working in a field/golf course doing maintenance/landscaping work potentially involving pesticide use; however, they were not doing this work during sample collection.

### Dust Levels: Trends and location differences

We detected 21 of the 25 analytes measured (Table 2). The majority of homes (93%) had at least three analytes detected in dust; 79% of the homes (n = 22) had at least six analytes detected and <1% (n = 2) of Salinas farmworker homes had up to 14 analytes detected in one sample. Cis- and trans-permethrin were the only insecticides detected in every home. Commonly detected OP pesticides included diazinon and chlorpyrifos. Diazinon was detected in 79% and 52% of the samples collected from Salinas farmworker and Oakland urban homes, respectively. Chlorpyrifos was detected in 55% and 36% of the samples collected from Salinas farmworker homes and Oakland urban homes, respectively. Other commonly detected analytes in samples collected from both locations included: allethrin (DF ≥ 80%), cypermethrin (DF ≥ 55%), and piperonyl butoxide (DF ≥ 86%). Detection frequencies were only significantly different between locations for chorthal-dimethyl, which was detected solely in Salinas farmworker homes.

**Table 2:** Limits of detection and summary statistics for pesticide dust concentrations (ng/g) in samples collected in 2006 from low-income farmworker and urban homes.^a,b^

	**Salinas farmworker homes** **(n = 29 samples collected from 15 homes)**								**Oakland urban homes** **(n = 25 samples collected from 13 homes)**						
		
	**LOD (ng/g)**	**DF**	**min**	**p25**	**p50**	**p75**	**p95**	**max**	**DF**	**min**	**p25**	**p50**	**p75**	**p95**	**max**
	
**Organophosphates**															
	
Diazinon	4	79	--	8.21	14.4	18	35.8	56.4	52	--	--	6.98	18.1	133	139
	
Chlorpyrifos	10	55	--	--	21.9	28	135	200	36	--	--	--	34.9	43.7	56.4
	
Malathion	10	7	--	--	--	--	52.2	70.8	12	--	--	--	--	877	1160
	
Tetrachlorvinphos	50	10	--	--	--	--	252	271	4	--	--	--	--	--	15.8
	
Diazinon-oxon	4	ND	--	--	--	--	--	--	4	--	--	--	--	--	4.73
	
Methidathion	10	ND	--	--	--	--	--	--	ND	--	--	--	--	--	--
	
Methyl Parathion	10	ND	--	--	--	--	--	--	ND	--	--	--	--	--	--
	
Phorate	10	ND	--	--	--	--	--	--	ND	--	--	--	--	--	--
	
															
	
**Pyrethroids**															
	
cis-permethrin	4	100	45.9	84.9	568	908	5930^c^	6300^c^	100	11.6	84.4	291	946	21600	26700
	
trans-permethrin	4	100	88.4	144	952	1380	9170^c^	9690^c^	100	18.4	166	504	1620	36400	46800
	
Allethrin^d^	10	83	--	18.4	57.1	129	652^c^	694	80	--	20.376	50.5	158	276	289
	
Cypermethrin^e^	20	55	--	--	230	918	4540	13500	64	--	--	587	1050	5990	13100
	
Bifenthrin	10	14	--	--	--	--	23.8	23.9	44	--	--	--	45	2050	2120
	
Sumithrin	10	24	--	--	--	--	591	807	8	--	--	--	--	104	116
	
Deltamethrin	250	17	--	--	--	--	3780	5590	12	--	--	--	--	13000	16300
	
Imiprothrin	50	7	--	--	--	--	253	2140	4	--	--	--	--	--	160
	
Prallethrin	2	ND	--	--	--	--	--	--	4	--	--	--	--	--	33.6
	
Esfenvalerate	50	3	--	--	--	--	--	66.5	ND	--	--	--	--	--	--
	
**Other**															
	
Piperonyl butoxide^f^	2	86	--	30.9	92.3	283	9060	9350	96	--	51.6	353	751	40300	46600
	
Chlorthal-dimethyl^g^	2	97	--	13.3	16.3	23.5	34.1	34.8	ND	--	--	--	--	--	--
	
Iprodione^h^	100	ND	--	--	--	--	--	--	ND	--	--	--	--	--	--

a. Two samples were obtained from each home in both locations except for one home in each location due to inadequate sample volume. b. Samples were collected from carpets or area rugs with the exception of three samples from two farmworker homes which were collected from furniture due to the absence of a carpet. c. Denotes that the reported concentration was observed in a furniture sample. d. Reported as the sum of two isomers (cis/trans) isomers. e. Reported as the sum of four isomers. f. Insecticide synergist. g. Phthalate herbicide h. Dicarboximide fungicide. Abbreviations and notation: LOD = limit of detection; DF = detection frequency (based on the number of samples obtained); ND or '--' indicates that analyte was not detected or detected <LOD so a summary statistic is not reported.

Median concentrations of diazinon, chlorpyrifos, permethrins, allethrin, and chlorthal-dimethyl were higher in Salinas farmworker homes compared to Oakland urban homes; however, only chlorthal-dimethyl concentrations were significantly different between locations. Analyses of location differences using pesticide loadings (ng/m^2^) did not change our findings (summary statistics for dust loadings are provided in the Additional files section; Additional file 2 Table A2).

Dust concentrations from furniture samples in two farmworker homes were comparable to those collected from carpets in other farmworker homes for frequently detected OPs (diazinon and chlorpyrifos), piperonyl butoxide, and chlorthal-dimethyl, while for frequently detected pyrethroids, concentrations were generally at the upper end of the distribution. We observed the same general pattern when using loadings. Maximum permethrin concentrations in farmworker homes were observed in furniture samples; however, the highest permethrin concentrations were observed in carpet samples from urban homes. The highest loading observed for cypermethrin was collected from a furniture sample; however, higher loadings were observed in carpet samples from urban homes. No location differences in pesticide concentrations or loadings were observed when we excluded furniture samples from our analysis.

Some of the less frequently detected analytes (e.g., tetrachlorvinphos, sumithrin) were detected with greater frequency in Salinas farmworker homes and at higher maximum concentrations than in Oakland urban homes. Conversely, the 95^th ^percentile and maximum concentrations for malathion and deltamethrin were higher among Oakland urban homes (Table 2).

Although not statistically significant, we generally observed higher dust concentrations in homes that reported recent pesticide use (i.e., within the last three months preceding the study) when pesticide containers were available to confirm the active ingredients. For example, in one home where bifenthrin had been applied less than a week before the first sample collection, concentrations were up to 200 times higher than the median concentration observed in other homes. Cypermethin was applied in one farmworker home, while imiprothrin was applied in two urban homes between the two sampling dates. For the farmworker home, cypermethrin dust concentrations were at the upper end of the distribution among other farmworker homes (between the 75^th ^and 95^th ^percentile concentrations reported). Imiprothrin was only detected in one of the urban homes which reported usage during the study; no other urban home had detectable imiprothrin levels indoors even though some of these households reported applying imiprothrin indoors prior to the study.

Concentrations in samples collected 5-8 days apart in the same home were positively and significantly correlated for the most frequently detected analytes (i.e., DF ≥ 50%), except allethrin; Spearman rank-order correlation coefficients ranged from 0.70 to 1.00 (p < 0.01) (Table 3).

**Table 3:** Spearman rank-order correlation coefficients for dust concentrations between the first and second collections for the most frequently detected analytes.^a^

Analyte	Salinas farmworker homes (n = 14)^b^	Oakland urban homes (n = 12) ^b^	All homes (n = 26)^c^
	Spearman rho		
	
Organophosphates			

Diazinon	0.88**	0.97**	0.92*

Chlorpyrifos	0.83**	--	--

Pyrethroids			

cis-permethrin	0.78**	1.00**	0.91**

trans-permethrin	0.70**	1.00**	0.90**

Allethrin	0.49	0.18	0.36

Cypermethrin	0.87**	0.89**	0.89**

Synergist Ingredient			

Piperonyl butoxide	0.77*	0.97**	0.89**

Phthalate Herbicide			

Chlorthal-dimethyl	0.78*	--	--

a. Only those homes for which we were able to measure analytes in both dust samples were included in these analyses. b. Spearman correlation coefficients are only provided when analyte DF≥50% in respective locations at each collection. c. Correlation coefficients for all homes are provided when analyte DF≥50% in both locations and collections. ** p ≤ 0.0001, * p < 0.01

### Temporal trends of chlorpyrifos and diazinon dust concentrations in Salinas farmworker homes after the U.S. EPA's residential phase-out

**Figure 1:**
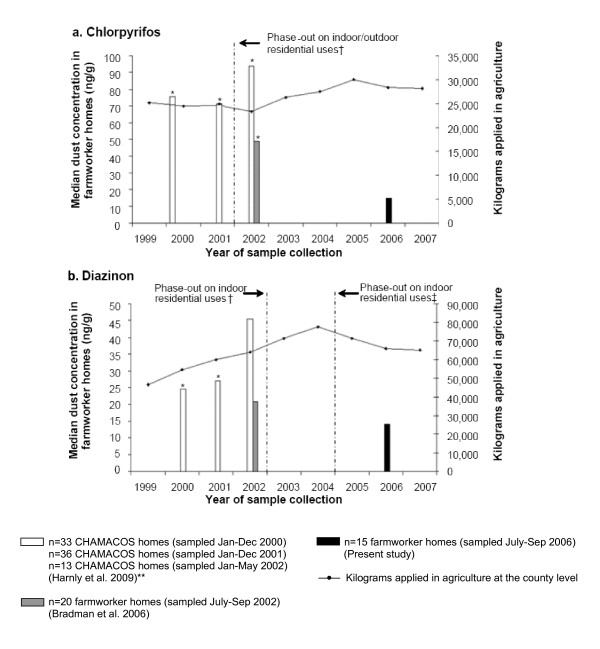
**Median chlorpyrifos and diazinon dust concentrations in samples from farmworker homes in the city of Salinas, CA by year of collection and kilograms applied (trendline) at the county-level (Monterey County) for agricultural purposes from 1999-2007**. † In December 2001 and 2002, residential products containing chlorpyrifos and diazinon, respectively were canceled. ‡Technical registrants were to buy back existing products from retailers by the end of December 2004. * Indicates that study had significantly higher dust concentrations compared to those observed in farmworker homes sampled in the present study (Wilcoxon Mann-Whitney tests, p < 0.05). ** CHAMACOS refers to the Center for the Health Assessment of Mothers and Children of Salinas longitudinal birth cohort study (Harnly et al. 2009).

As noted earlier, residential formulations of chlorpyrifos and diazinon were voluntarily phased-out by the end of 2001 and between 2002 and 2004 [24,25], respectively. However, agricultural use of chlorpyrifos and diazinon in Monterey county generally increased from 1999-2007 (trendline in Figure 1), most notably for diazinon. Non-agricultural uses in Monterey County (i.e., applications for landscape maintenance, public health, commodity fumigation, rights-of-way, and structural pest control) for both of these OP pesticides was a small fraction (<6%, ≈ 20-1,500 kgs/yr) of agricultural use between 1999 and 2005, and declined further through 2006-2007 (Table 4). As shown in Figure 1, median dust concentrations of chlorpyrifos and diazinon were 70-80% and 40-50% lower, respectively, in the present farmworker homes sampled in 2006 compared to samples collected between 2000 and 2002 from farmworker homes in the same Salinas zip codes. Chlorpyrifos dust concentrations differed significantly between the present study and each of the previous studies (Wilcoxon Mann-Whitney tests, p < 0.05). Diazinon concentrations were significantly lower in the present study compared to CHAMACOS households sampled prior to 2002 (p < 0.05).

**Table 4:** Kilograms of diazinon and chlorpyrifos applied in Monterey county from 1999-2007 for non-agricultural applications.^a,b^

Year	Diazinon (kgs)	Chlorpyrifos (kgs)
1999	717	1519

2000	841	678

2001	1301	355

2002	1094	760

2003	1076	101

2004	217	18

2005	96	54

2006	6	3

2007	2	<0.5

a. Pounds applied at the county level are reported by year by the California Department of Pesticide Regulation in their Pesticide Use Reporting (PUR) database. Pounds reported in the database were converted to kilograms. b. Non-agricultural applications refer to uses such as landscape maintenance, public health, commodity fumigation, rights-of-way, and structural pest control applications by licensed applicators. Source: California Department of Pesticide Regulation Pesticide Use Regulation Database. Available at: http://www.cdpr.ca.gov/docs/pur/purmain.htm

### Estimated non-dietary ingestion intake and hazard quotients

For the 14 detected pesticides with available RfDs, none of the hazard quotients for resident children's non-dietary dust ingestion exceeded 1.0 (i.e., estimated intake did not exceed the RfD, Table 5).

**Table 5:** Summary statistics on the estimated intake and hazard quotients (HQ) for all study children.^a^

					Select Summary Statistics for HQs based on 54 dust samples from farmworker and urban children			
					
Analyte	RfD (mg/kg/dy)^b^	# samples with concentration >LOD	Range of Intake (mg/kg/day)^c^		p50	p75	p95	Max
Organophosphates			**Min**	**Max**				

diazinon	0.0002	36	--	7.0 × 10^-07^	2.5 × 10^-04^	4.3 × 10^-04^	1.6 × 10^-03^	3.5 × 10^-03^

chlorpyrifos	0.00003	25	--	1.1 ×10^-06^	--	4.9 × 10^-03^	2.3 × 10^-02^	3.8 × 10^-02^

malathion	0.07	5	--	4.9 × 10^-06^	--	--	2.8 × 10^-06^	7.0 × 10^-05^

tetrachlorvinphos	0.04	4	--	1.5 × 10^-06^	--	--	2.2 × 10^-05^	3.9 × 10^-05^

Pyrethroids								

cis-permethrin^d^	0.25	54	5.4 × 10^-08^	1.3 × 10^-04^	9.7 × 10^-06^	2.0 × 10^-05^	8.4 × 10^-05^	5.1 × 10^-04^

trans-permethrin^d^	0.25	54	8.6 × 10^-08^	2.2 × 10^-04^	1.7 × 10^-05^	3.2 × 10^-05^	1.6 × 10^-04^	8.9 × 10^-04^

cypermethrin	0.06	32	--	6.4 × 10^-05^	2.5 ×10^-05^	8.4 × 10^-05^	4.8 × 10^-04^	1.1 × 10^-03^

bifenthrin	0.015	15	--	1.1 × 10^-05^	--	4.4 × 10^-06^	2.6 × 10^-05^	7.5 × 10^-04^

sumithrin	0.0007	9	--	3.7 × 10^-06^	--	--	3.2 × 10^-03^	5.2 × 10^-03^

deltamethrin	0.0033	8	--	9.0 × 10^-05^	--	--	6.7 × 10^-03^	2.7 × 10^-02^

prallethrin	0.025	1	--	1.9 × 10^-07^	--	--	--	7.4 × 10^-06^

esfenvalerate	0.02	1	--	4.4 × 10^-07^	--	--	--	2.2 × 10^-05^

Others								

chlorthal-dimethyl	0.01	28	--	1.8 × 10^-07^	3.0 × 10^-06^	7.2 × 10^-06^	1.5 × 10^-05^	1.8 × 10^-05^

piperonyl butoxide	0.16	49	--	2.2 × 10^-04^	5.0 × 10^-06^	1.7 × 10^-05^	2.5 × 10^-04^	1.4 × 10^-03^

a. A hazard quotient (HQ) was calculated as the ratio of the potential dust intake to the respective analyte reference dose (RfD). The HQ was only calculated for those analytes for which an RfD was available. b. Chronic population adjusted doses (cPADs) were used as the reference dose for chlorpyrifos (cPAD for children and females 13-50 years of age) and deltamethrin. Sources: IRIS database http://cfpub.epa.gov/ncea/iris/index.cfm?fuseaction=iris.showSubstanceList and EPA's Pesticide Registration Status: http://www.epa.gov/opp00001/reregistration/status.htm. c. Intake was calculated by multiplying the toxicant dust concentration by an ingestion rate of 0.10 g/day (100 mg/day) then dividing by the child-specific body weight (kg). d. The RfD available for "permethrin" was used for each individual isomer in our calculations. Notation: '--' Value is not reported since dust concentrations were less than the limit of detection.

## Discussion

We detected several pesticides in most homes, including OP pesticides previously phased-out for residential uses, pyrethroids, and the pesticide synergist piperonyl butoxide (PBO). Biological exposure metrics for these pesticides are relatively transient and highly variable, typically reflecting recent exposures [35]. However, consistent with other studies [15,36], we found that dust serves as a stable matrix and indicator of potential indoor exposure for some pesticides. The high correlations observed in dust concentrations from samples collected 5-8 days apart suggests that, for some pesticides, measurements in house dust may be relatively stable indicators of potential indoor exposure over this time frame. To our knowledge, this is the first study to evaluate the correlation of concentrations within homes for several pesticides over a short sampling period.

Although the detection frequency for chlorpyrifos and diazinon was higher in Salinas than Oakland, we did not observe statistically significant differences in pesticide concentrations or loadings between locations. This is notable given that >28,000 and 65,000 kgs of chlorpyrifos and diazinon, respectively, were applied for agricultural purposes in Monterey County in 2006 (Additional File 1 Table A1) and minimal applications (65 kgs and 3 kgs of chlorpyrifos and diazinon, respectively) occurred in Alameda County. Previously, we showed a significant correlation with local agricultural use and chlorpyrifos dust concentrations (but not diazinon) for homes throughout the Salinas Valley [13]. Mapping of dust concentrations and agricultural use suggests that chlorpyrifos dust concentrations are higher in the center of the Valley (south of the city of Salinas), where agricultural use is higher [13]. Farmworker homes in the present study were from the city of Salinas where the impact of drift from agricultural applications may have been lower. Additionally, our small sample size may have prevented us from observing significant differences in concentrations between locations for these OP pesticides as well as other analytes.

Malathion was not frequently detected in homes from either location; however, higher levels were observed in urban homes. This pesticide is used in agriculture and is also registered for use in home gardens, as a building perimeter treatment, as a wide-area spray for mosquitoes, and by prescription for head-lice control [37]. However, no parents reported treating their children for lice or using it themselves in their gardens. The main county uses for this OP pesticide in 2006 in the urban region were landscape maintenance and structural pest control [38]. These applications were reported more than 25 km away from the nearest study home, thus it is not readily apparent why higher levels were observed in urban homes although it should be noted that we only sampled a small number of homes.

We generally observed significantly lower house dust concentrations of chlorpyrifos and diazinon in the present study compared to levels measured in dust from homes located in the same zip codes sampled between 2000 and 2002 [10,13], suggesting that indoor concentrations in the city of Salinas are decreasing despite continued agricultural use in the area. In New York City, air concentrations for these OP pesticides in low-income homes also significantly decreased between 2001 and 2004 [26]. The temporal declines in indoor concentrations reported here and in the New York City study may reflect the decreasing usage of these OP pesticides for home or structural applications per the U.S. EPA's residential phase-out. Nonetheless, despite declining concentrations indoors, detection of these OP pesticides, especially in Oakland where there was little agricultural or structural use, underscores their persistence indoors.

Compared to other studies in farmworker populations (Table 6), we observed lower median concentrations for chlorpyrifos [10,13,17,22,39,40] and diazinon [10,13,40]. These farmworker studies generally reported a wider range of concentrations for these two OP pesticides and collected dust samples prior to the residential phase-out. One study by Curl et al. [22] reported a wider range of diazinon concentrations, but comparable median concentrations (10 ng/g). Although malathion was not frequently detected in our farmworker homes, a wider range of concentrations was reported in previous farmworker studies (Table 6) [10,22,40]. To our knowledge, only one other study has reported OP pesticide concentrations in low-income urban homes [41]. This study reported higher median concentrations for chlorpyrifos and diazinon in low-income urban housing units in Boston, MA. Homes in this study were sampled just after or during the residential phase-out of chlorpyrifos and diazinon, respectively (between July 2002 and August 2003).

**Table 6:** Dust concentrations for select organophosphorous pesticides and pyrethroids from select U.S. farmworker studies (ng/g).^a^

Author	Population	Location	Collection method	Sampling Dates	Pesticides	LOD (ng/g)^b^	DF%	n	Range (ng/g)	Median	Mean (SD)
Harnly et al. 2009^c^	Farmworkers (CHAMACOS longitudinal birth cohort)	Salinas Valley, CA	HVS3	2000-2002	Organophosphates:						
					Chlorpyrifos	2	91	177-197	2.9-7850	74	NR
					Diazinon	2	86		4.7-2870	26	
											
					Pyrethroids:						
					cis-Permethrin	5	98		16-168000	344	
					trans-Permethrin	5	98		146-265000	467	
											
					Others:						
					Chlorthal-dimethyl	2	98		2.3-271	22	

Bradman et al. 2006^d^	Farmworkers	Salinas Valley, CA	HVS3	June-September 2002	Organophosphates:						
					Chlorpyrifos	2	95	20	<LOD-1200	49	NR
					Diazinon		100		4-810	21	
					Malathion		20		<LOD-480	NR	
											
					Pyrethroids:						
					cis-Permethrin		100		13-2900	150	
					trans-Permethrin		100		22-5800	230	
											
					Others:						
					Chlorthal-dimethy		100		6.5-110	31	

Rothlein et al. 2006	Farmworkers	Hood River, OR	HVS3	Summer 1999	Chlorpyrifos	10	92	26	<LOD-1200	130	200(240)
					Diazinon	10	77		<LOD-720	310	310(230)
					Malathion	10	81		<LOD-1400	180	380(400)

Curl et al. 2002	Agricultural Workers	Yakima Valley, Washington State	Nilfisk vacuum cleaner	June-September 1999	Chlorpyrifos	150	26	156	<LOD-2560	50	NR
					Diazinon	170	3.8		<LOD-770	10	
					Malathion	160	15		<LOD-1030	40	

Fenske et al. 2002^e^	Ag (at least one family member employed as an orchard applicator (APP) or farmworker (FW))	Central Washington State (major tree fruit production region)	HVS3	May-July 1995	Chlorpyrifos	LOQ: 13-27(varied batch to batch)	APP: 100 FW: 100	APP: 49 FW: 12	APP: 10-2600 FW: 70-560	APP: 370 FW: 250	APP: 550(580) FW: 270(180)

Simcox et al. 1995	Farmers (F), Farmworkers (FW)^e^	Wenatchi area (eastern Washington State)	HVS3	Jan-July 1992	Chlorpyrifos	LOD: 20 ng/mL LOQ: 17 ng/g	F: 96 FW:100	F: 26 FW: 22	F: <LOD-3585 FW: 40-2180	F: 372 FW: 172	F: 506 FW: 338 SD not reported

a. Other studies may have measured additional analytes; only those relevant to the ones measured in our study are included. b. Unless otherwise indicated, limits of detection (LODs) are in ng/g. In some cases a limit of quantitation (LOQ) was reported instead of an LOD. c. Other analytes were also measured, but detection frequencies were <50%. Analytes included: malathion, methidathion, and iprodione. Minimum value reported in the "Range" column is the lowest quantified concentration. d. Other analytes were also measured, but detection frequencies were <50%. Analytes included allethrin, bifenthrin, cypermethrin, deltamethrin, esfenvalerate, sumithrin, and iprodione. Malathion had a detection frequency <50% in this study, but respective information is presented for comparison with other farmworker studies. e. Also included a reference population, but information is not provided in this table. SD = Standard deviation; NR: Value not reported; <LOD: Indicates that value reported was below the limit of detection or not detected.

Pyrethroids were detected in house dust in several study homes. Similar to low-income urban housing units in Boston, MA [41], pyrethroids and PBO were detected in higher concentrations and used more frequently in our study homes compared to other pesticides. This finding is consistent with the fact that pyrethroid insecticide formulations for residential applications have largely replaced OP pesticide residential formulations [42,43]. Although over 19,000 kgs of permethrin were applied in Monterey County in 2006 for agricultural purposes [44], we did not observe significant differences in permethrin concentrations (or loadings) between locations. Allethrin and cypermethrin were also widely detected in most homes. Our findings suggest that home use likely contributed to the presence of pyrethroid pesticides in house dust since pyrethroids were commonly used indoors and negligible to no agricultural applications took place at the county level (except for permethrin). It is also possible that structural pest control applications influenced indoor detection of certain pyrethroids in some homes. For example, it is estimated that ~80% of the non-agricultural cypermethrin use reported in Alameda County in 2006 was for structural pest control [38]. The presence of pyrethroids in house dust is also consistent with their physical and chemical properties, including high octanol:water partition coefficient values (log K_ow _> 4.0) and low vapor pressures (Additional File 1 Table A1). To our knowledge, only two studies [10,13] have measured pyrethroid dust concentrations in farmworker homes. Similar to the present study, permethrins were the most frequently detected pyrethroids indoors. Median cis- and trans-permethrin concentrations in our farmworker homes were higher than those observed in a previous study [10].

The detection of chlorthal-dimethyl in all Salinas farmworker homes and none of the Oakland urban homes is consistent with other Salinas Valley studies showing an association between agricultural use and house dust contamination [13] and a positive correlation between outdoor and indoor air concentrations [10]. This herbicide had relatively high agricultural use (~ 33,970 kgs) in the Salinas Valley and is not found in home-use pesticides. Chlorthal-dimethyl also has a high log K_ow _value and low vapor pressure (Additional File 1 Table A1), and may be bound to particulate matter at room temperature.

Over 16,000 kgs of malathion and iprodione were used in 2006 for agricultural applications (Additional File 1 Table A1); however, they were not commonly detected in farmworker homes from the city of Salinas. For some of these pesticides, e.g., iprodione, LODs were higher than for other analytes. Other factors including physico-chemical properties, e.g., high vapor pressure and low log K_ow _values (≤3), may have resulted in lower detection frequencies. These pesticides were also not frequently detected in dust samples from our previous study in the city of Salinas [10].

This study has several limitations. Location differences in pesticide dust levels have been reported previously when using loadings rather than concentrations [21]; however, our small sample size limits statistical power and may have prevented us from observing statistically significant differences between locations for concentrations and/or loadings. Additionally, although homes with insufficient sample mass were demographically similar to those with adequate sample mass, exclusion of these homes may have introduced some bias and prevented us from detecting a difference in pesticide concentrations and/or loadings between locations. We also focused on low-income homes and thus the results may not be generalizable to other populations. Although estimated intakes for select pesticides were below EPA RfDs (i.e., HQ <1.0), it should not be concluded that intakes below RfDs are "acceptable" or free of any health risks. For example, recent studies have identified mechanisms of OP pesticide toxicity that were not considered in defining current U.S. EPA RfDs (e.g., suppressed expression of serotonin transporter genes) [45]. Moreover, RfDs do not account for differences in vulnerability to pesticide toxicity due to genetic factors, such as paraoxonase (PON1) polymorphisms [46]. Additionally, our intake calculations for pesticides do not account for other exposure pathways (e.g., inhalation or diet); nor did we consider that some children could have pica or other behaviors that could increase or decrease intake. Although we surveyed participants on their usage of pesticides indoors, we were not always able to corroborate whether formulation ingredients were present at high concentrations as the pesticide containers were not always available to confirm the active ingredients. Lastly, children in the homes sampled are clearly exposed to multiple indoor contaminants and our hazard evaluation does not account for exposure to complex mixtures.

## Conclusions

Studies of contaminants in low-income homes, including our study, have been limited in sample size and, often, selection of participants has not been random. In addition, collection methods, analytical techniques, analytes measured, and timing of data collection differ. To our knowledge, only one other study has assessed indoor dust concentrations of pyrethroids in low-income homes in an urban setting [41]. Nonetheless, the results from these studies indicate that low-income children are potentially exposed to a mixture of pesticides. Agricultural pesticide use may contribute to additional exposures to some pesticides in rural areas; historical or current residential use is also likely to contribute to ongoing exposures. Although children's non-dietary intake did not exceed U.S. EPA RfDs for select pesticides, this does not ensure that children are free of any health risks as RfDs have their own limitations, and the children may be exposed indoors via other pathways. The frequent pesticide use reported among participating households in this and previous studies of low-income homes [18,19,41] and high detection of several home-use pesticides in house dust suggests there is a need to educate families on the potential health impacts of pesticide use and effective integrated pest management strategies to control pests and reduce exposures to household occupants [42]. Particular at-risk populations are those living in households with poorer housing quality, where there may be greater needs for pest control [18,19].

Additional research is needed to quantify exposures and potential health effects from these compounds, particularly frequently used pesticides such as pyrethroids. Such research should consider the complex mixture of chemicals found in indoor environments, include both environmental and biomonitoring measurements to assess cumulative exposures, and consider exposures in homes of different socioeconomic status.

## List of Abbreviations

CDPR: California Department of Pesticide Regulation; cPAD: Chronic Adjusted Population Dose; DF: Detection frequency; GEE: Generalized Estimating Equation; HQ: Hazard Quotient; HVS3: High Volume Small Surface Sampler; K_oc_: Water:Organic Carbon Partition Coefficient; K_ow_: Octanol:Water Partition Coefficient; LOD: Limit of Detection; OP: Organophosphorous; PBO: Piperonyl butoxide; PDI: Potential daily intake; PON1: Paraoxonase 1; PUR: Pesticide Use Reporting; RfD: Reference Dose; SRS: Surrogate Recovery Standard; U.S. EPA: United States Environmental Protection Agency.

## Competing interests

The authors declare that they have no competing interests.

## Authors' contributions

**LQA: **conceived of the study; participated in the design, coordination, and implementation of all study field activities; conducted the statistical analysis; and drafted the manuscript; **AB: **conceived of the study; participated in the design, coordination, and implementation of all study field activities; and helped to draft the manuscript; **MN: **responsible for laboratory analysis of dust samples and quality assurance and control, and helped to draft the laboratory analysis section of the manuscript; **MEH: **provided assistance with previous CHAMACOS data used in the analysis of temporal trends of phased-out pesticides and helped to draft the manuscript; **AH: **contributed to the statistical phase and helped to draft the data analysis and results section of the manuscript; **TEM: **helped to draft the manuscript; **JF: **responsible for cleaning the data and providing feedback on the manuscript; **BE: **conceived of the study; participated in the design, coordination, and implementation of all study field activities; and helped to draft the manuscript. All authors read and approved the final manuscript.

## Supplementary Material

Additional file 1 **Table A1**. Select analyte chemical and physical properties and amounts applied in 2006 for agricultural and non-agricultural purposes in the counties where our homes were sampled. This file contains information on select chemical and physical properties for the analytes measured in dust samples as well as information on their usage at the county level in the year in which samples were collected. Click here for file

Additional file 2 **Table A2**. Summary statistics for dust loadings (ng/m2) in samples collected in 2006 from low-income urban and farmworker homes. This file contains information on select summary statistics on analyte dust loadings in the homes sampled. Click here for file
