# Supplementary material for: Pesticides in house dust from urban and farmworker households in California: an observational measurement study
Source: Environ Health. 2011 Mar 16;10:19. doi: 10.1186/1476-069X-10-19 (PMC3071308; doi:10.1186/1476-069X-10-19)
Supplement: Additional file 1 — Table A1. Select analyte chemical and physical properties and amounts applied in 2006 for agricultural and non-agricultural purposes in the counties where our homes were sampled. This file contains information on select chemical and physical properties for the analytes measured in dust samples as well as information on their usage at the county level in the year in which samples were collected. [file 1476-069X-10-19-S1.PDF]

Table A1. Select analyte chemical and physical properties and amounts applied in 2006 for agricultural and non-agricultural purposes in the counties where our homes were sampled.[†]

Analyte	CAS no.	Molecular Weight (g/mol)	log K_{ow} (octanol:water partition coefficient)	Vapor Pressure (Pa)	Henrys Law Constant (Pa-m^3/mol)	Amount applied for agricultural and non-agricultural purposes in 2006 (kgs) [a,b]	
						Monterey County (agricultural community) Ag (Non-ag) use	Alameda County (urban community) Ag (Non-ag) use:
**Organophosphates**							
Chlorpyrifos[‡]	2921-88-2	350.6	5.0	2.7×10^{-03}	2.94×10^{-01}	28335 (3)	65 (3)
Diazinon[‡]	333-41-5	304.4	3.8	1.2×10^{-02}	1.11×10^{-02}	65872 (6)	3 (<2)
Malathion	121-75-5	330.4	2.4	2.4×10^{-02}	4.96×10^{-04}	16900 (56)	<1 (581)
Methidathion	950-37-8	302.3	2.2	4.5×10^{-04}	7.30×10^{-04}	3861 (NA)	NA
Methyl Parathion	298-00-0	263.2	2.9	2.0×10^{-04}	$8.51 \times 10^{+01}$	94 (NA)	NA (11)
Phorate	298-02-2	260.4	3.6	8.5×10^{-02}	4.46×10^{-01}	276	NA
Tetrachlorvinphos	22248-79-9	366.0	3.5	5.6×10^{-06}	1.82×10^{-04}	NA	NA
**Pyrethroids**							
Allethrin[‡]	584-79-2	302.4	5.0	1.6×10^{-04}	6.18×10^{-02}	NA	NA (<1)
Bifenthrin	82657-04-3	422.9	>6	2.4×10^{-05}	1.01×10^{-01}	400 (29)	1 (212)
Cypermethrin[‡]	52315-07-8	416.3	6.6	4.1×10^{-07}	4.26×10^{-02}	618 (38)	NA (404)
Deltamethrin	52919-63-5	505.2	6.2	2.0×10^{-06}	5.07×10^{-01}	<1 (16)	NA (67)
Esfenvalerate	66230-04-4	419.9	6.2	2.0×10^{-07}	4.15×10^{-02}	1317 (<1)	1 (<1)
Imiprothrin	72963-72-5	318.4	2.9	1.8×10^{-06}	not found	NA	NA
Permethrin[‡]	52645-53-1	391.3	6.5	2.9×10^{-06}	1.93×10^{-01}	19486 (308)	15 (478)
Prallethrin	23031-36-9	300.4	4.5	1.3×10^{-05}	1.00×10^{-02}	NA	NA
Sumithrin	26002-80-2	350.5	7.5	1.9×10^{-05}	6.89×10^{-01}	<1(<1)	NA (<1)
**Others**							
Chlorthal-dimethyl[‡]	1861-32-1	332.0	4.4	3.3×10^{-04}	2.23×10^{-01}	33969 (53)	NA
Iprodione	36734-19-7	330.0	3.0	5.0×10^{-07}	3.14×10^{-04}	17095 (249)	2 (121)
Piperonyl Butoxide[‡]	51-03-6	207.3	4.8	3.5×10^{-05}	9.02×10^{-06}	165 (16)	NA (71)

† Unless otherwise specified by a footnote, main source used was HSDB(2001); Available at:http://toxnet.nlm.nih.gov/cgi-bin/sis/htmlgen?HSDB

‡ Analyte detected in more than 50% of the samples in at least one location.

a. Agricultural usage is reported followed by non-agricultural usage in parentheses. Usage reported is at the county level; Salinas farmworker homes are located in Monterey County, and Oakland urban homes are located in Alameda County. Source: California Department of Pesticide Regulation Pesticide Use Reporting (PUR) database (2006):Monterey county (Salinas, CA): http://www.cdpr.ca.gov/docs/pur/pur06rep/chemcnty/monter06_ai.pdf; Alameda county (Oakland, CA): http://www.cdpr.ca.gov/docs/pur/pur06rep/chemcnty/alamed06_ai.pdf.

b. Non-agricultural uses include applications for landscape maintenance, rights of way, commodity fumigation, structural pest control by licensed applicators, and public health.

c. Source: IUPAC Pesticides Database; Available at: http://sitem.herts.ac.uk/aeru/iupac/1474.htm.
