# Supplementary material for: Pesticides in house dust from urban and farmworker households in California: an observational measurement study
Source: Environ Health. 2011 Mar 16;10:19. doi: 10.1186/1476-069X-10-19 (PMC3071308; doi:10.1186/1476-069X-10-19)
Supplement: Additional file 2 — Table A2. Summary statistics for dust loadings (ng/m2) in samples collected in 2006 from low-income urban and farmworker homes. This file contains information on select summary statistics on analyte dust loadings in the homes sampled. [file 1476-069X-10-19-S2.PDF]

Table A2. Summary statistics for analyte dust loadings (ng/m^2) in samples collected in 2006 from low-income farmworker and urban homes.[a,b]

	Salinas farmworker homes (n=29 samples collected from 15 homes)							Oakland urban homes (n=25 samples collected from 13 homes)						
	DF	min	p25	p50	p75	p95	max	DF	min	p25	p50	p75	p95	max
**Organophosphates**														
Diazinon	79	--	8.64	23	73	358	742	52	--	--	9.48	185	1470	2190
Chlorpyrifos	55	--	--	27.7	68.5	2310	2640	36	--	--	--	60.3	551	646
Malathion	7	--	--	--	--	50.3	70.5	12	--	--	--	--	877	2690
Tetrachlorvinphos	10	--	--	--	--	846	3320	4	--	--	--	--	--	20.8
Diazinon-oxon	ND	--	--	--	--	--	--	4	--	--	--	--	--	12
Methidathion	ND	--	--	--	--	--	--	ND	--	--	--	--	--	--
Methyl Parathion	ND	--	--	--	--	--	--	ND	--	--	--	--	--	--
Phorate	ND	--	--	--	--	--	--	ND	--	--	--	--	--	--
**Pyrethroids**														
cis-permethrin	100	88.6	375	816	4110	14200	14500	100	24.4	348	768	7200	32200	69000
trans-permethrin	100	187	721	1570	6550	20300[c]	21500	100	46.6	624	1620	12600	56400	116000
Allethrin[d]	83	--	34.1	128	565	1020	1120[c]	80	--	68	129	523	1740	2980
Cypermethrin[e]	55	--	--	438	2340	16300	42300	64	--	--	1650	6710	19100	24400
Bifenthrin	14	--	--	--	--	37.1[c]	83.3	44	--	--	--	71.1	16000	39200
Sumithrin	24	--	--	--	--	4130	8860	8	--	--	--	--	204	550
Deltamethrin	17	--	--	--	--	7290	9500	12	--	--	--	--	140000	208000
Imiprothrin	7	--	--	--	--	210	7690	4	--	--	--	--	--	6330
Prallethrin	ND	--	--	--	--	--	--	4	--	--	--	--	--	362
Esfenvalerate	3	--	--	--	--	--	223	ND	--	--	--	--	--	--
**Other**														
Piperonyl butoxide	86	--	101	359	1240	10600	30100	96	--	144	678	14000	48600	149000
Chlorthal-dimethyl	97	--	23.4	46.1	71.9	175	537	ND	--	--	--	--	--	--
Iprodione	ND	--	--	--	--	--	--	ND	--	--	--	--	--	--

a. Two samples were obtained from each home in both locations except for one home in each location due to inadequate sample volume. b. Samples were collected from carpets or area rugs with the exception of three samples from two farmworker homes which were collected from furniture due to the absence of a carpet in each home. c. Denotes that the reported concentration was observed in a furniture sample.
d. Loading calculated after summing concentrations for two isomers (cis-/trans-) isomers. e. Loading calculated after summing concentrations for four isomers. Abbreviations and notation: DF=detection frequency, ND=not detected. '---' Indicates that concentration used to calculate loading was below the limit of detection or not detected thus, a loading is not reported.
